# Metformin Attenuates the Exacerbation of the Allergic Eosinophilic Inflammation in High Fat-Diet-Induced Obesity in Mice

**DOI:** 10.1371/journal.pone.0076786

**Published:** 2013-10-24

**Authors:** Marina Ciarallo Calixto, Letícia Lintomen, Diana Majoli André, Luiz Osório Leiria, Danilo Ferreira, Camilo Lellis-Santos, Gabriel Forato Anhê, Silvana Bordin, Richardt Gama Landgraf, Edson Antunes

**Affiliations:** 1 Department of Pharmacology, Faculty of Medical Sciences, State University of Campinas (UNICAMP), Campinas, São Paulo, Brazil; 2 Department of Physiology and Biophysics, Institute of Biomedical Sciences, University of São Paulo, São Paulo, SP, Brazil; 3 Department of Biologic Sciences, Federal University of São Paulo, São Paulo, SP, Brazil; University Hospital Freiburg, Germany

## Abstract

A positive relationship between obesity and asthma has been well documented. The AMP-activated protein kinase (AMPK) activator metformin reverses obesity-associated insulin resistance (IR) and inhibits different types of inflammatory responses. This study aimed to evaluate the effects of metformin on the exacerbation of allergic eosinophilic inflammation in obese mice. Male C57BL6/J mice were fed for 10 weeks with high-fat diet (HFD) to induce obesity. The cell infiltration and inflammatory markers in bronchoalveolar lavage (BAL) fluid and lung tissue were evaluated at 48 h after ovalbumin (OVA) challenge. HFD obese mice displayed peripheral IR that was fully reversed by metformin (300 mg/kg/day, two weeks). OVA-challenge resulted in higher influx of total cell and eosinophils in lung tissue of obese mice compared with lean group. As opposed, the cell number in BAL fluid of obese mice was reduced compared with lean group. Metformin significantly reduced the tissue eosinophil infiltration and prevented the reduction of cell counts in BAL fluid. In obese mice, greater levels of eotaxin, TNF-α and NOx, together with increased iNOS protein expression were observed, all of which were normalized by metformin. In addition, metformin nearly abrogated the binding of NF-κB subunit p65 to the iNOS promoter gene in lung tissue of obese mice. Lower levels of phosphorylated AMPK and its downstream target acetyl CoA carboxylase (ACC) were found in lung tissue of obese mice, which were restored by metformin. In separate experiments, the selective iNOS inhibitor aminoguanidine (20 mg/kg, 3 weeks) and the anti-TNF-α mAb (2 mg/kg) significantly attenuated the aggravation of eosinophilic inflammation in obese mice. In conclusion, metformin inhibits the TNF-α-induced inflammatory signaling and NF-κB-mediated iNOS expression in lung tissue of obese mice. Metformin may be a good pharmacological strategy to control the asthma exacerbation in obese individuals.

## Introduction

Obesity is strongly associated with metabolic syndrome, hypertension, dyslipidemia and hyperglycemic tendencies that are represented at the molecular level by insulin resistance (IR) [1]. Obesity and weight gain are considered risk factors for asthma exacerbations [2]. Obese asthmatic patients exhibit worse asthma control [3] and do not respond properly to standard therapy as lean asthmatic patients [4,5]. Studies show that IR accounts for most of the obesity-associated asthma risk in children and adults [2,6]. Animal studies have provided additional support for a relationship between obesity and asthma [7]. Airway hyperresponsiveness is enhanced in genetically obese mice [8]. Ovalbumin (OVA) challenge in previously sensitized *ob/ob* mice (that are obese as a result of a genetic leptin deficiency) aggravates the pulmonary resistance [9] and eosinophilic inflammation [10]. Moreover, high-fat diet-induced obesity was recently shown to exacerbate the lung eosinophilic inflammation through enhanced eosinophil trafficking from bone marrow to lung tissues, and delayed their transit through the airway epithelium into the airway lumen [11]. 

Obesity-associated changes in immunomodulatory factors play an important role in the pathogenesis of both IR and asthma. TNF-α undergoes up-regulation by the presence of these pathological conditions and acts directly in regulating adipocyte fat accumulation, playing a role in IR development [12,13]. Obese mice lacking TNF-α display protection against IR [14]. TNF-α is able to induce a great diversity of cellular responses via modulation of expression of different genes through activation of nuclear transcription factors, such as nuclear factor (NF)- κB [15]. Obesity is also associated with increased inducible NOS (iNOS) expression and subsequent NO overproduction [16]. The molecular mechanisms for iNOS gene transcription occur mainly via activation of the transcription factor NF-kB [17]. Pharmacological inhibition of NO or iNOS gene deletion decreases diet-induced adiposity and improves the insulin signaling in skeletal muscle, indicating a strong relationship between IR and iNOS expression in rodent models [16,18]. Moreover, NO plays a pivotal role in the eosinophil infiltration into the airway of asthmatic individuals and animals [19,20]. Accordingly, the allergic pulmonary inflammation and eosinophil infiltration are significantly reduced in iNOS knockout mice [21]. Treatment with the selective iNOS inhibitor aminoguanidine inhibits the influx of inflammatory cells induced by the allergen [22].

Metformin is the first line oral anti-hyperglycemic drug for patients with type 2 diabetes mellitus [23]. Metformin is well tolerated and highly efficient in reducing blood glucose in insulin resistant individuals, which is mainly attributed to reductions in hepatic glucose output and increases in peripheral glucose uptake [24]. At the cellular level, metformin activates AMP-activated protein kinase (AMPK), an energy sensor involved in the regulation of cellular metabolism that is activated by increases in the intracellular AMP levels [25]. Activation of AMPK inhibits inflammatory processes in different models [26] such as colitis [27], cystic fibrosis [28], autoimmune encephalomyelitis [29] and LPS-induced lung inflammation [30]. AMPK has also been identified as a counter-regulator of macrophage inflammatory function, promoting macrophage polarization towards an anti-inflammatory phenotype [31]. Metformin was recently shown to reduce the murine allergic airway inflammation and remodeling via activation of AMPK [32]. Since AMPK activation reverses IR and inhibits different inflammatory processes [26-31], the present study investigated if metformin was capable to attenuate the exacerbation of allergic eosinophilic inflammation in obese mice. We tested the hypothesis that metformin inhibits the TNF-α-induced inflammatory signaling and nuclear factor (NF)-κB-mediated iNOS expression in lung tissue of obese mice.

## Materials and Methods

### Animals and Diet

All animal procedures and experimental protocols are in accordance with the Ethical Principles in Animal Research adopted by the Brazilian College for Animal Experimentation (COBEA) and were approved by the institutional Committee for Ethics in Animal Research/State University of Campinas (CEEA-UNICAMP, protocol 2008/1496-1). Animals were housed on a 12-h light–dark cycle and fed for 10 weeks with either a standard chow diet (70% carbohydrate; 20% protein, 10% fat) or a high-fat diet that induces obesity (29% carbohydrate, 16% protein, 55% fat) [11].

### Sensitization Procedure to OVA Challenge

**Figure 1:**
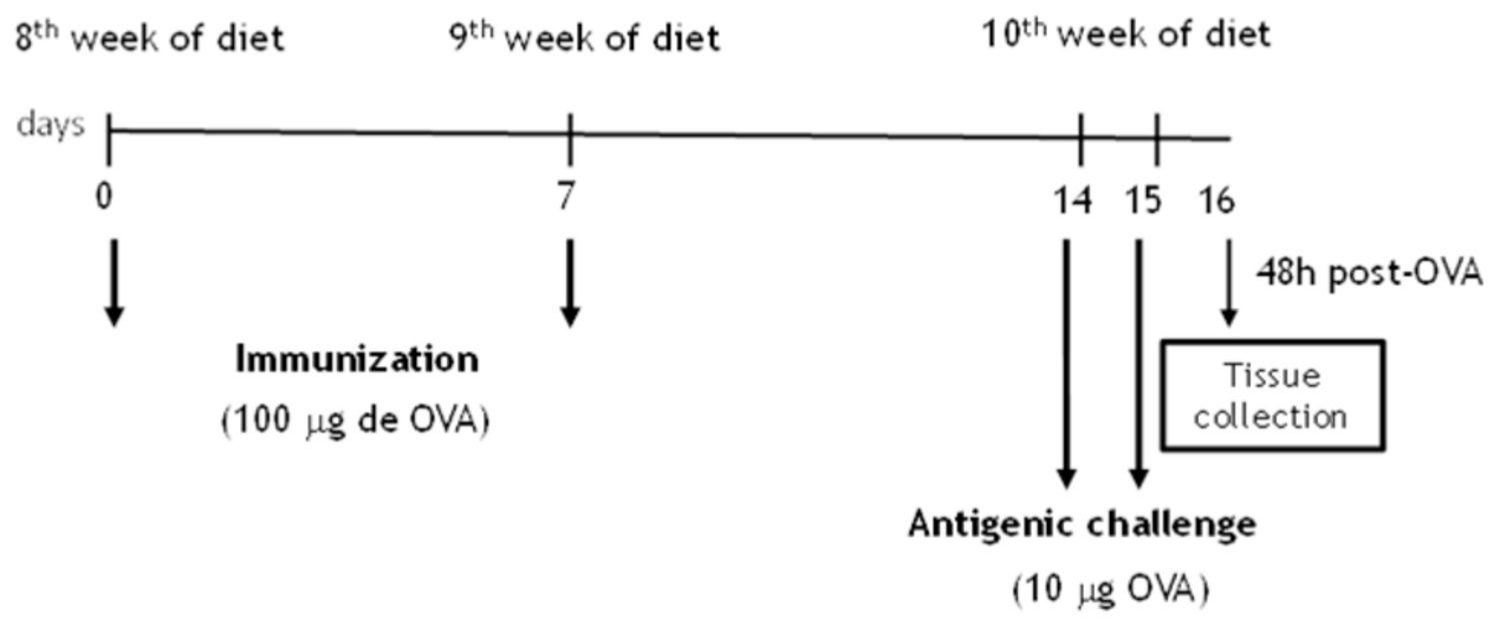
Schematic representation of ovalbumin (OVA) immunizations and challenges.

Lean and obese mice were actively sensitized with a subcutaneous injection (0.4 ml) of 100 μg of OVA (grade V; Sigma-Aldrich Co., St. Louis, MO) mixed with 1.6 mg Al(OH)_3_ in 0.9% NaCl (Day zero). One week later (Day 7), mice received a second subcutaneous injection of 100 μg OVA (0.4 ml). On days 14 and 15, mice were intranasally challenged with OVA (10 μg/50 μl) twice a day. At 48 h after the first challenge, the bronchoalveolar lavage (BAL) was performed (for details, see Figure 1). Lungs were also collected for morphological studies, Western blot and CHIP assays. 

### Treatments

**Figure 2:**
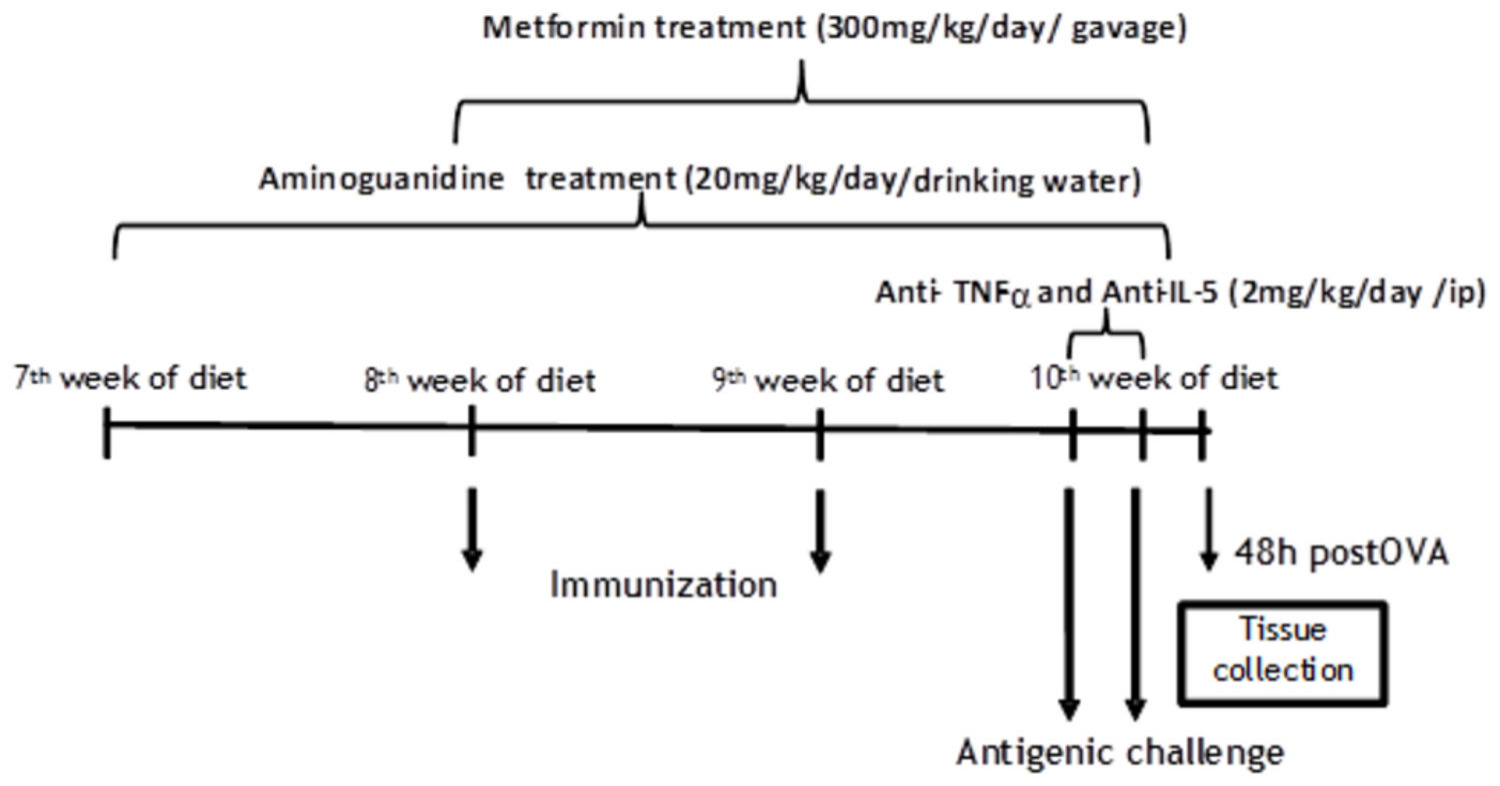
Schematic representation for treatments with metformin, aminoguanidine, anti-TNF-α and anti-IL-5 in ovalbumin (OVA)-sensitized and challenged mice.

Lean and obese mice were treated with vehicle (water) or metformin (300 mg/Kg/day) by gavage for two weeks [33] or aminoguanidine (20 mg/kg/day) given in the drinking water for 3 weeks [34]. For TNF-α and IL-5 blockade, lean and obese mice were treated with neutralizing monoclonal antibody (mAb; Biolegend, San Diego, USA) to mouse TNF-α or IL-5 (2 mg/kg each, given intraperitoneally at days 14 and 15 and 1 h before the first OVA challenge). Control mice received the isotype immunoglobulin (IgG) in the same doses. Figure 2 details the time-course treatments for metformin, aminoguanidine, anti-TNF-α mAb and anti-IL-5 mAb.

### BAL Fluid

The lungs were washed by flushing phosphate-buffered saline (PBS). The PBS was instilled through the tracheal cannula in 5-aliquots of 300 µl. The fluid recovered after each instillation was centrifuged (500 × *g*, 10 min, 4°C), and BAL fluid supernatant stored at -80°C. The cell pellet was resuspended in 200 µl of PBS and total (Neubauer) and differential (Diff-Quick stain) cell counts were done. 

### Morphometrical Analysis

Lungs were perfused via the right ventricle with 10 ml PBS to remove residual blood, immersed in 10% phosphate buffered formalin for 24 h and then kept in 70% ethanol until embedding in paraffin. Tissues were sliced (5 μm sections) and stained with hematoxylin/eosin for light microscopy examination. Morphometrical analysis was performed using a Nikon DXM 1200c digital camera, and Nikon NIS – Elements AR 2.30 Software. For each different staining, the area of positivity was measured in mm^2^ for 10 bronchioles per slide (or the maximum number of bronchioles in each slide). 

### Measurement of Cytokines and NOx Levels

TNF-α, IL-5 and eotaxin-1 were measured in BAL fluid using commercially available DuoSet ELISA kits, following the instructions of the manufacturer (R & D, Minneapolis, USA). The NOx levels (sum of nitrate and nitrite) in BAL fluid were determined using commercially available kits (Cayman Chemical, Ann Arbor, MI, USA). 

### Western Blotting for iNOS, Phosphorylated AMPK and Phosphorylated ACC

Lung tissues were homogenized in an SDS lysis buffer with a Polytron PTA 20S generator (model PT 10/35; Brinkmann Instruments, Inc., Westbury, NY) and centrifuged. Protein concentrations in supernatants were determined by the Bradford assay, and equal amount of protein from each sample (50 µg) was treated with Laemmli buffer containing dithiothreitol (100 mM). Samples were heated in a boiling water bath for 10 min and resolved by SDS-PAGE. Electrotransfer of proteins to nitrocellulose membrane was performed for 60 min at 15 V (constant) in a semi-dry device (Bio-Rad, Hercules, CA, USA). Nonspecific protein binding to nitrocellulose was reduced by pre-incubating the membrane overnight at 4°C in blocking buffer (0.5% non-fat dried milk, 10 mM Tris, 100 mM NaCl, and 0.02% Tween 20). Detection using specific antibodies, HRP-conjugated secondary antibodies, and luminol solution was performed. Anti-iNOS, anti-phospho-AMPKα1/α2 (Thr 172) and anti-phosphoACC (ser 79) antibodies were obtained from AbCam Technology (Cambridge, England, UK), and anti GAPDH was from Santa Cruz Biotechnologie (Santa Cruz, CA, USA). Densitometry was performed using the Scion Image Software (Scion Corporation, Frederick, MD). Densitometry was performed using the Scion Image software (Scion Corporation, Frederick, MD) and results represented as the ratio of the density of the primary antibodies band to the density of the GAPDH band.

### Chromatin Immunoprecipitation (ChIP) Assay

Lung fragments for the ChIP assay were processed as previously described [35]. After DNA shearing, samples were precleared for 1 h at 4° C with protein A-Sepharose saturated with salmon sperm DNA. An aliquot of 10 µl was collected as input. The remaining supernatants were immunoprecipitated with protein A-Sepharose and 2 µg of anti-p65 NF-κB antibody (Abcam, Cambridge, MA, USA). In parallel, one sample was incubated only with protein A-Sepharose to generate the negative control. DNA extracted from the Sepharose pellets was subjected to cross-linking reversal and purification using phenol-chloroform. DNA samples were amplified for detection of the iNOS gene. The mouse iNOS gene was amplified by real-time PCR. The sequences of the primers were sense 5’GCAAGCACTTTACCAACTGAGCC3’ and antisense 5’CTAGCACATCCTGCCAGGGTCC3´ (annealing at 62° C). To check the primer specificity (by estimated product length), reaction products were resolved in an ethidium bromide-agarose gel. The p65 NF-κB binding was calculated after normalization to the input of each sample.

### Insulin Tolerance Test (ITT)

After 6 h fasting, systemic insulin sensitivity was analyzed by the Insulin Tolerance Test (ITT). Briefly, tail blood samples were collected before (0 min) and at 5, 10, 15, 20, 25 and 30 min after an intraperitoneal injection of 1.00 U/Kg of regular insulin (Novolin R, NovoNordisk, Bagsvaerd, Denmark). Glucose concentrations were measured using a glucometer (ACCUCHEK Performa; Roche Diagnostics, Indianapolis, IN, USA) and the values were used to calculate the constant rate for blood glucose disappearance (K_ITT_), which based on the linear regression of the neperian logarithm of glucose concentrations obtained from 0 to 30 min of the test. Kitt was calculated using the formula 0.693/(t_1/2_)×−1×100 [36].

### Statistical Analysis

Data are presented as the means ± SEM of n experiments. The program Instat (GraphPad software) and the SAS System for Windows (version 8.02) were used for statistical analysis. Two-way repeated measures ANOVA was used to analyze the insulin tolerance test data. One-way ANOVA followed by Tukey test was performed to analyze the other data. A value of p<0.05 was accepted as significant.

## Results

### Body Weight, Epididymal Fat Mass and Insulin Tolerance Test

**Figure 3:**
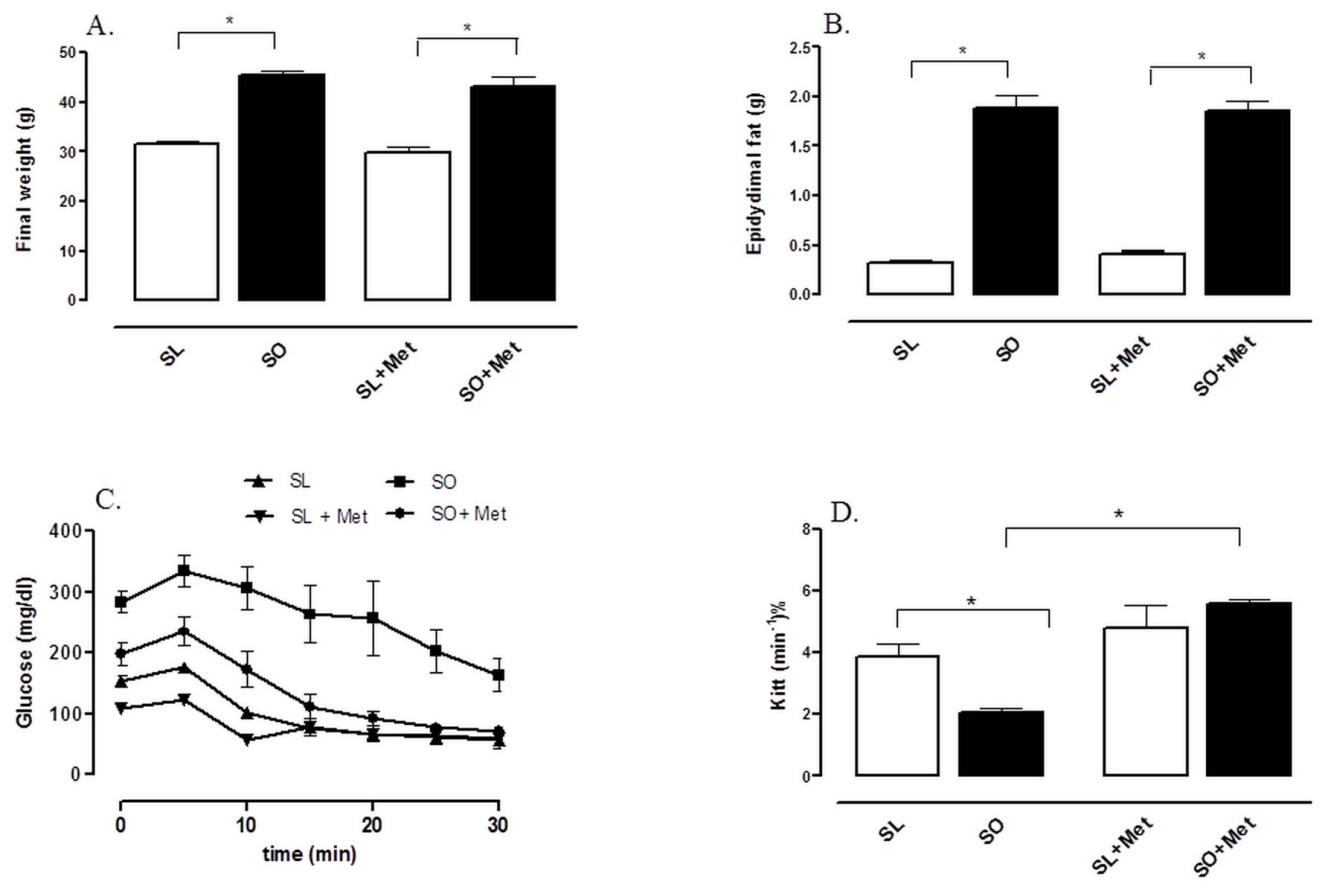
Effect of metformin treatment on body weight (A), epididymal fat mass (B), insulin tolerance test (C) and glucose decay rate (K_itt_; %/min) (D). Male C57BL6/J mice were fed with either a standard chow diet (lean) or a high-fat diet (obese) during 10 weeks. Metformin (300mg/kg/dia) was given by gavage for the last two weeks. Each column represents the mean ± SEM (n = 6) for mice sensitized lean treated with vehicle (SL), sensitized obese treated with vehicle (SO), sensitized lean treated with metformin (SL + Met) and sensitized obese treated with metformin (SO + Met). *p<0.05 (SO vs SL); #p<0.05 (SO + Met vs SO).

The high-fat-fed mice exhibited significant increases in body weight and epididymal fat mass compared with lean mice. Treatment with metformin (300 mg/kg/day, two weeks) did not significantly affect these parameters (Figure 3 A and B).

To test if treatment with metformin reduces obesity-induced IR, we evaluated the blood glucose levels before and at 5 to 30 min after administration of recombinant human insulin (1 IU/kg), and calculated the constant rate for glucose disappearance (K_ITT_). In lean mice, blood glucose levels rapidly (10 min) decreased to baseline after insulin administration (Figure 3C). In contrast, in obese mice, the fall in glucose levels after insulin administration was of slow onset, and glucose levels remained higher than lean group throughout the measurement period (Figure 3C). A reduced K_ITT_ value was found in obese mice, indicating resistance to insulin action in these animals (Figure 3D). Treatment with metformin prevented the reduction of K_ITT_ in obese mice, showing a protection against obesity-induced IR (Figure 3D). Metformin did not significantly affect the blood glucose levels and K_ITT_ value in lean mice.

### Effect of Metformin on Pulmonary Cell Recruitment in BAL Fluid

We initially carried out control experiments in BAL fluids of (1) non-sensitized mice instilled with PBS, (2) non-sensitized mice instilled with OVA and (3) OVA-sensitized mice instilled with PBS. Our data showed that cells in BAL fluid from the non-sensitized mice instilled with PBS were >99% mononuclear cells, as observed in both lean and obese groups (data not shown, n = 5). In the non-sensitized mice instilled with OVA, leukocytes in BAL fluid consisted mainly of mononuclear cells, with few neutrophils (4% and 11% for the lean and obese groups, respectively; n = 5). Similarly, in OVA-sensitized mice instilled with PBS, leukocytes in BAL fluid consisted of mononuclear cells, with few neutrophils (2% and 6% for the lean and obese groups, respectively; n = 5). There were virtually no eosinophils in both of these control groups (non-sensitized challenged with OVA or OVA-sensitized instilled with PBS). 

**Figure 4:**
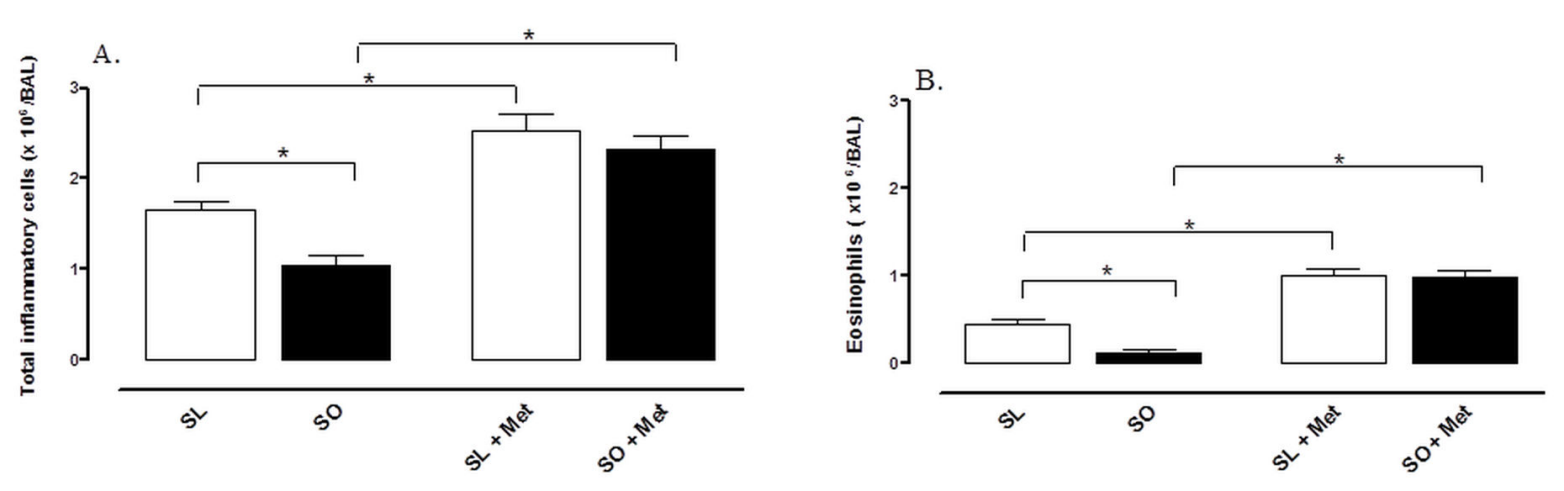
Effect of metformin treatment (300 mg/kg/day, two weeks) on the number of total inflammatory cells (A) and eosinophils (B) in bronchoalveolar lavage (BAL) fluid at 48 h following intranasal challenge with ovalbumin in sensitized mice. Each column represents the mean ± SEM (n = 10) for mice sensitized lean treated with vehicle (SL), sensitized obese treated with vehicle (SO), sensitized lean treated with metformin (SL + Met) and sensitized obese treated with metformin (SO + Met). *p<0.05.

In lean mice, OVA challenge in previously sensitized animals markedly increased the number of total cells and eosinophils in BAL fluid (1.7 × 10^6^ ± 0.05 and 0.6 × 10^6^ ± 0.05 respectively) compared with non-sensitized group (0.9 × 10^6^ ± 0.08 and 0.00 × 10^6^ ± 0.00 respectively; p<0.05). However, in obese mice, the infiltration of total cells and eosinophils in BAL fluid was significantly lower in comparison with lean group (Figure 4). 

In BAL fluid of lean mice, treatment with metformin promoted an increase in the counts of total cells and eosinophils (Figure 4). In addition, in obese mice, metformin fully prevented the reductions in total cell and eosinophil counts (Figure 4). 

The number of neutrophils in BAL fluid of obese mice was also reduced compared with lean group, and metformin treatment prevented such reduction (Table 1). Regarding the mononuclear cells, there was no difference between lean and obese groups, but metformin treatment increased the number of these cells in both groups, although the increase was higher in the obese mice (Table 1). 

**Table 1:** Number of neutrophils and mononuclear cells in bronchoalveolar lavage (BAL) fluid at 48 h following intranasal challenge with ovalbumin in sensitized mice, treated or not with metformin (300 mg/kg/day, two weeks).

**Group**	**Neutrophils** (x 10^6^cels/BAL)	**Mononuclears** (x 10^6^cels/BAL)
SL	0.51 ± 0.1	0.62 ± 0.06
SO	0.18 ± 0.02*	0.70 ± 0.18
SL + Met	0.46 ± 0.04	0.94 ± 0.11 #
SO + Met	0.41 ± 0.04	1.29 ± 0.13^◆^†

Data were obtained from sensitized controls animals (SL), sensitized obese (SO), sensitized controls treated with metformin (SL + Met), sensitized obese treated with metformin (SO + Met). Each column represents the mean ± SEM for n = 10-15. *p <0.05 compared to SC; # p <0.05 compared with SC; ◆ p <0.05 compared with SO, † p <0.05 compared with SC + Met.

### Effect of Metformin on Pulmonary Cell Recruitment in Lung Tissue

**Figure 5:**
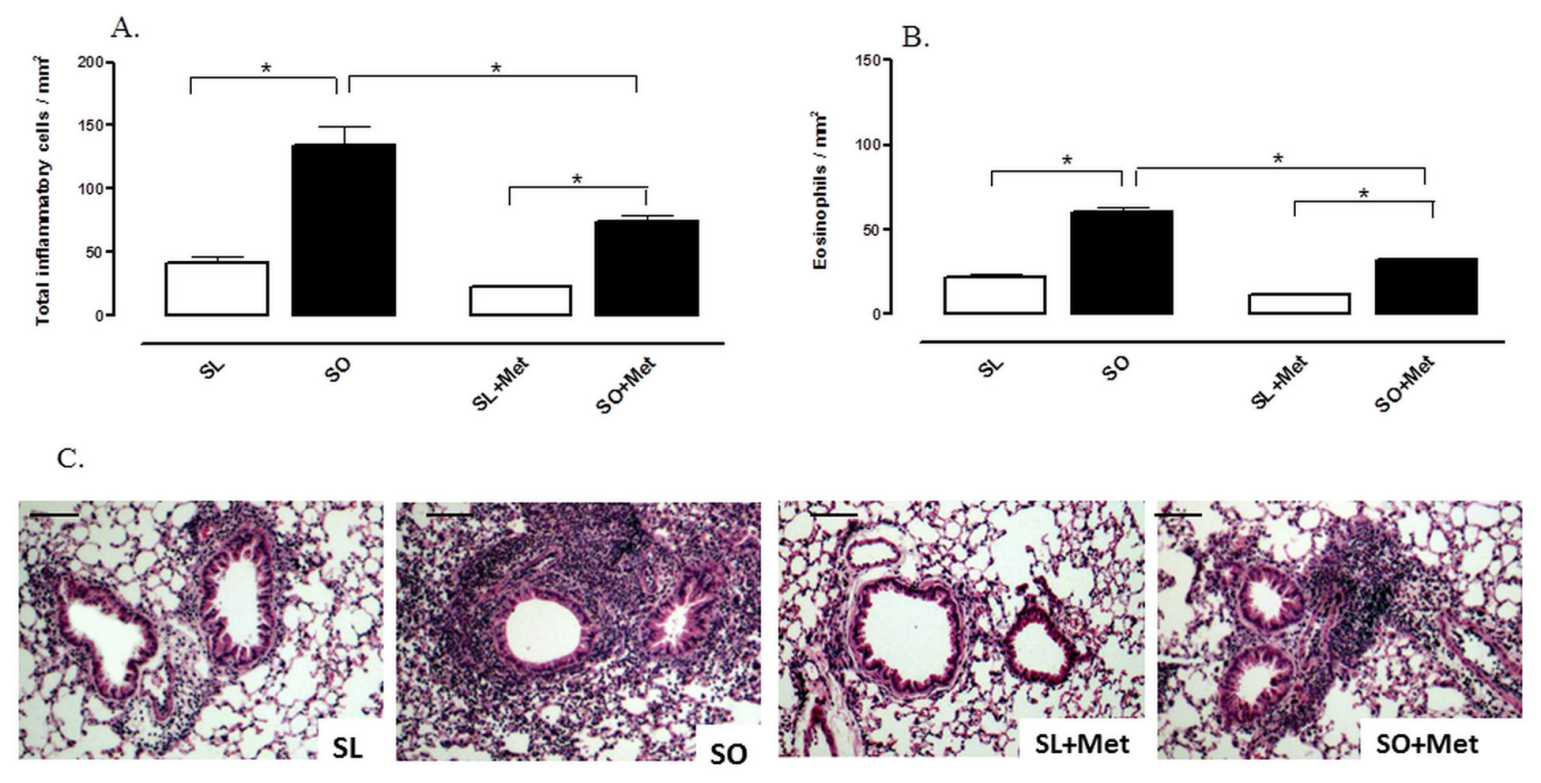
Effect of metformin treatment (300 mg/kg/day, two weeks) on the number of total inflammatory cells (A) and eosinophils (B) in lung connective tissue surrounding the bronchial and bronchiolar segments at 48 h following intranasal challenge with ovalbumin in the sensitized mice. Representative high-power fields of bronchiolar structures from the following groups: mice sensitized lean treated with vehicle (SL), sensitized obese treated with vehicle (SO), sensitized lean treated with metformin (SL + Met) and sensitized obese treated with metformin (SO + Met). Panel C shows representative images of lung histology for the four experimental groups. Haematoxylin–eosin, high magnification (bar represents 20 µm). Each column represents mean ± SEM (n = 6) of the number of cells mm^2^. *p<0.05.

The morphometrical analysis of the lung tissue of OVA-challenged mice showed that infiltration of total inflammatory cells and eosinophils in the peribronchiolar region was markedly greater in obese compared with lean mice (Figure 5A, B and C). Metformin treatment significantly attenuated the infiltration of total inflammatory cells and eosinophils in the lung tissue of obese mice, without significantly affecting the pattern of cell infiltration in lean group (Figure 5A, B and C).

### Effect of Metformin on Levels of IL-5, Eotaxin and TNF-α

**Figure 6:**
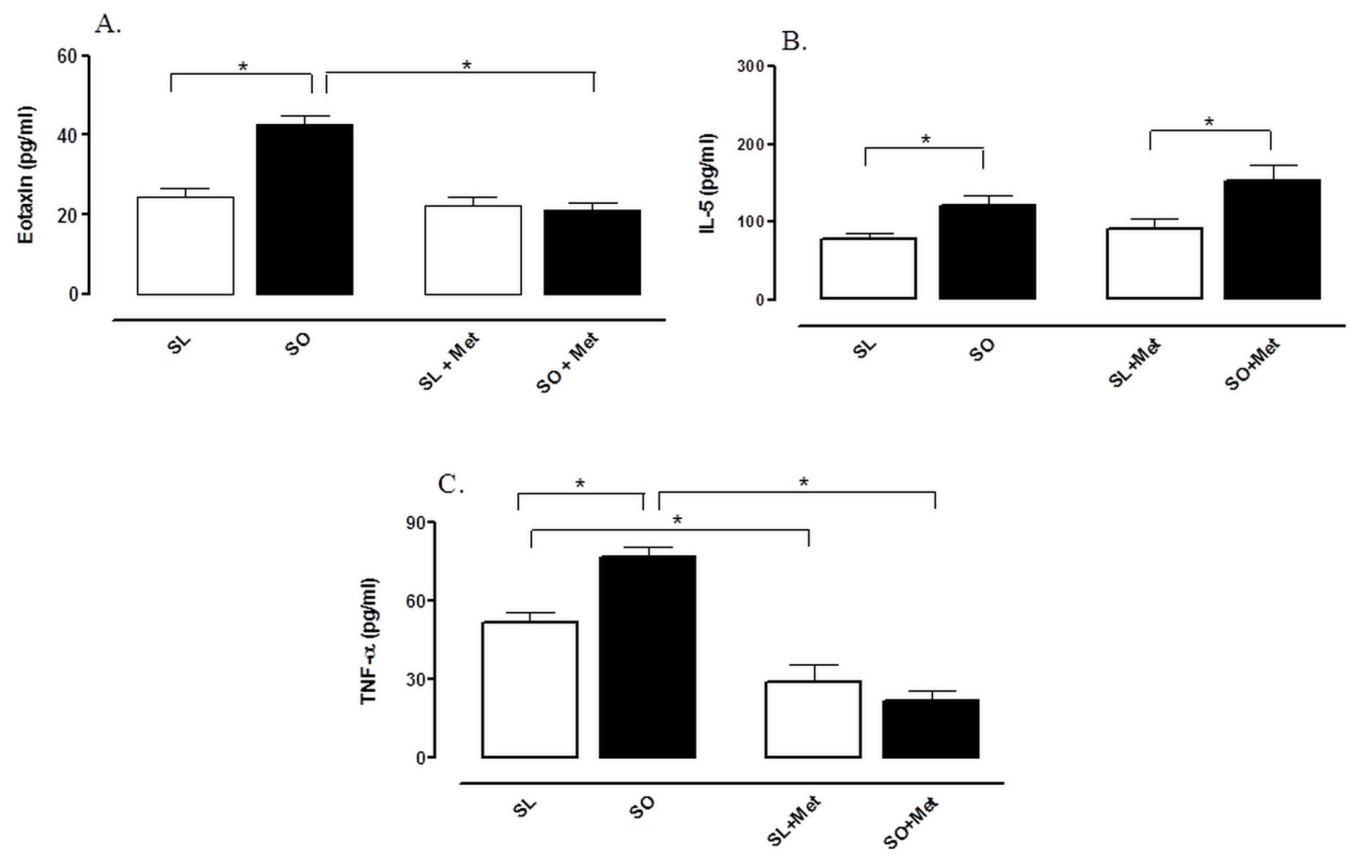
Effect of metformin treatment (300 mg/kg/day, two weeks) on the levels of eotaxin (A), IL-5 (B) and TNF-α (C) in bronchoalveolar lavage (BAL) fluid at 48 h following intranasal challenge with ovalbumin in sensitized mice. Each column represents the mean ± SEM (n = 6-10) for mice sensitized lean treated with vehicle (SL), sensitized obese treated with vehicle (SO), sensitized lean treated with metformin (SL + Met) and sensitized obese treated with metformin (SO + Met). * p<0.05.

The levels of eotaxin and IL-5 in BAL fluid after OVA challenge were significantly greater in obese compared with lean mice. Metformin normalized the levels of eotaxin (but not of IL-5) in obese mice without affecting the levels in lean group (Figure 6A and B). The levels of TNF-α were also significantly greater in BAL fluid of obese compared with lean mice. Metformin reduced the TNF-α levels in lean mice, and prevented its enhanced levels in obese group (Figure 6C).

### Levels of NO_x_, iNOS Expression and NF-kB Binding to the iNOS Promoter in the Lung Tissue

**Figure 7:**
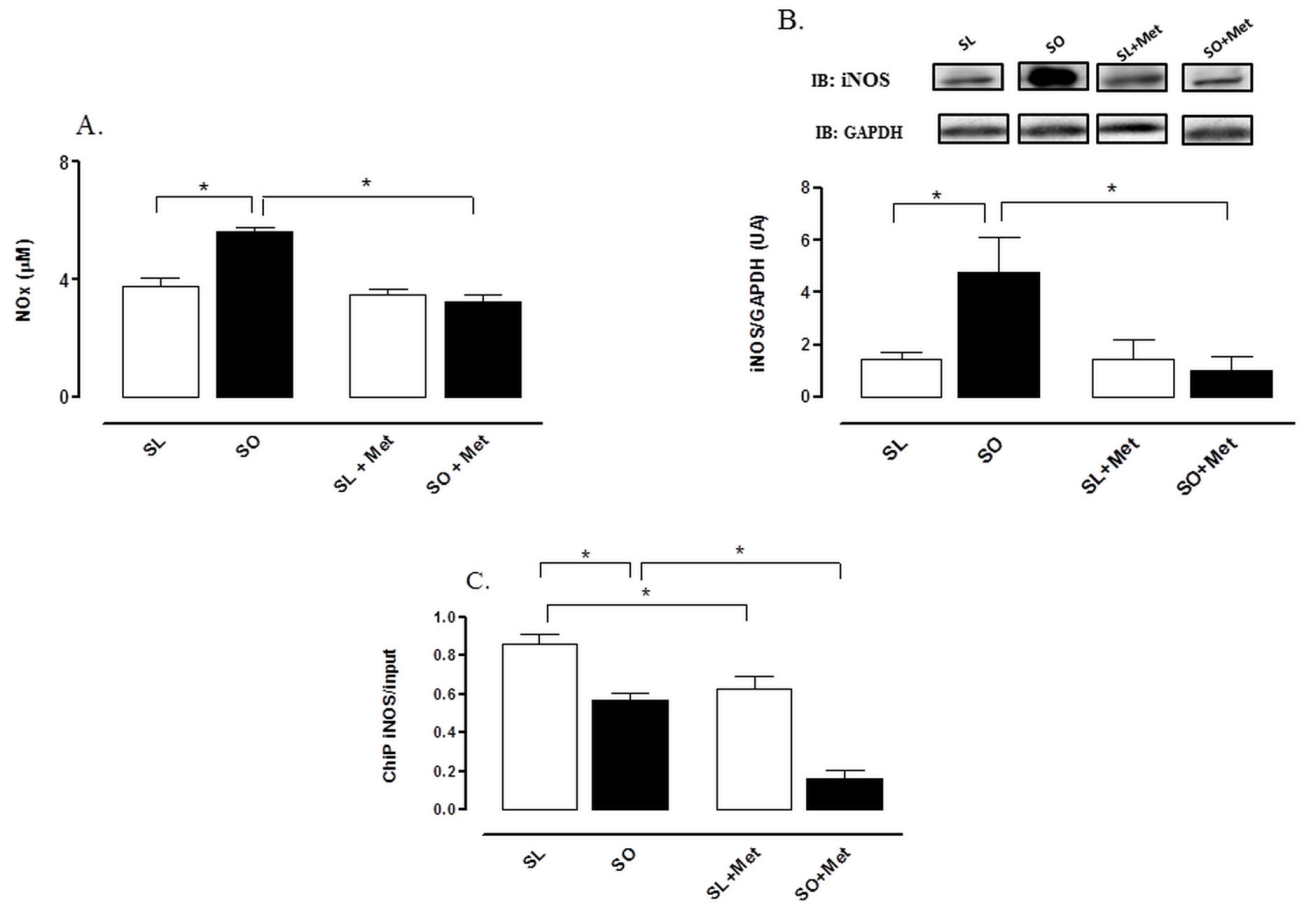
Effect of metformin treatment (300 mg/kg/day, two weeks) on the levels of nitric oxide metabolites (NOx) in bronchoalveolar lavage (BAL) fluid (A), and inducible NOS expression (iNOS) (B) and NF-kB binding to iNOS gene (C) in lung tissue (B) at 48 h following intranasal challenge with ovalbumin in sensitized mice. NF-kB binding to iNOS gene was evaluated in fragments of lungs using chromatin immunoprecipitation (ChIP) using an anti-p65 NF-kB antibody (the iNOS gene was amplified from the ChIP samples and normalized to the respective input). Each column represents the mean ± SEM (n = 6) for mice sensitized lean treated with vehicle (SL), sensitized obese treated with vehicle (SO), sensitized lean treated with metformin (SL + Met) and sensitized obese treated with metformin (SO + Met) mice. The membranes probed with iNOS antibody were normalized with GAPDH * p<0.05.

Obese sensitized animals showed greater levels of NOx in the BAL fluid after OVA challenge compared with lean group, which was normalized by metformin treatment. Metformin had no effect on NOx levels in lean group (Figure 7A).

The iNOS expression was markedly higher in the lung tissue of obese compared with lean group (Figure 7B). Metformin nearly restored to control levels the iNOS expression in the obese group, without affecting the iNOS expression in lean group (Figure 7B).

In addition, chromatin immunoprecipitation (ChIP) assays revealed that metformin treatment produced a much larger inhibition of the binding of NF-κB subunit (p65) to the iNOS promoter in the lung tissue of obese in comparison with lean mice (Figure 7C).

### Effects of Aminoguanidine on Airway Inflammatory Responses

**Figure 8:**
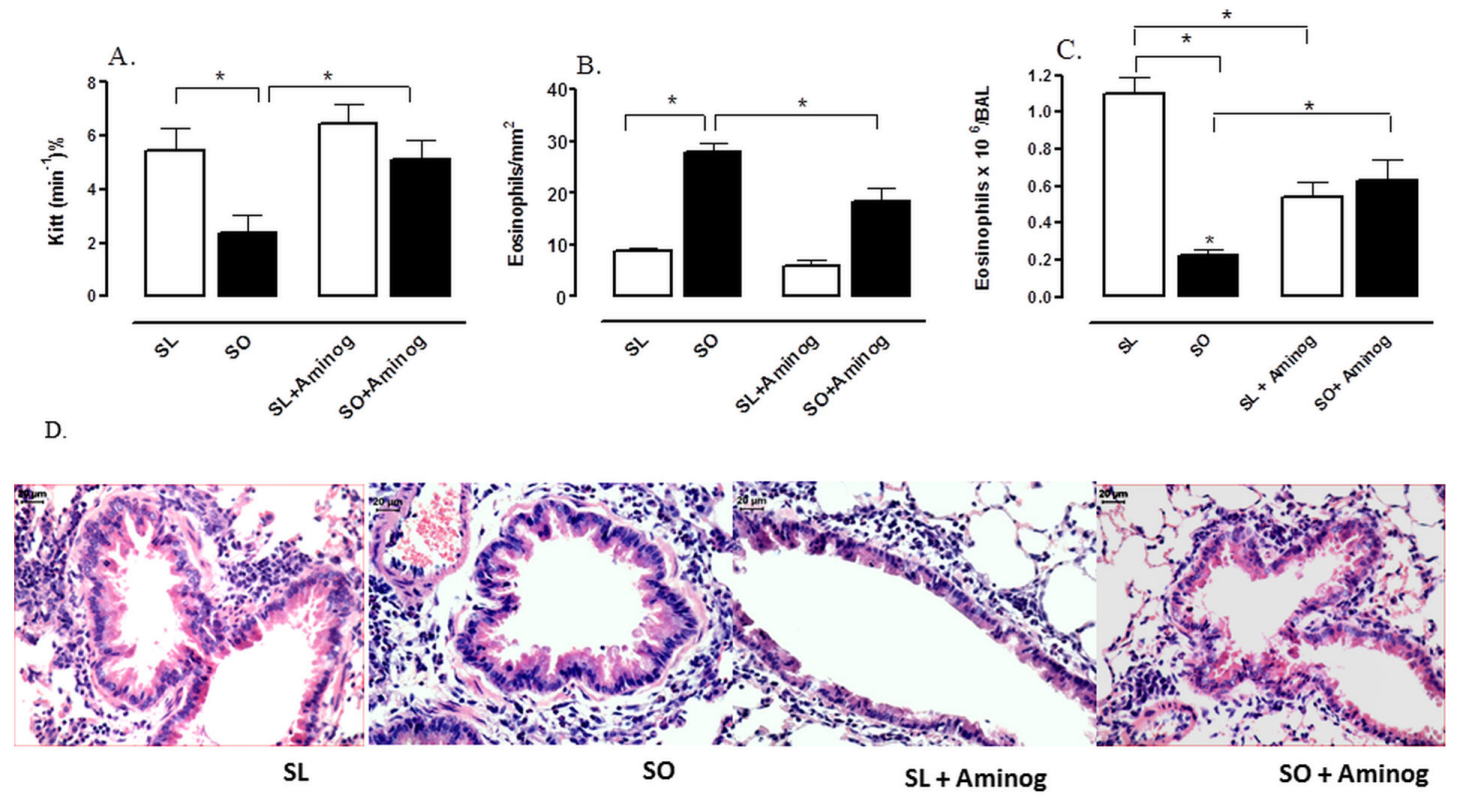
Effect of aminoguanidine (20mg/kg) on glucose decay rate (K_itt_; %/min) (A), number of eosinophils in the lung connective tissue surrounding the bronchial and bronchiolar segments (B) and bronchoalveolar lavage (BAL) fluid (C) at 48 h following intranasal challenge with ovalbumin in sensitized mice. Representative high-power fields of bronchiolar structures from the following groups: mice sensitized lean treated with vehicle (SL), sensitized obese treated with vehicle (SO), sensitized lean treated with amoniguanidine (SL + Aminog) and sensitized obese treated with amoniguanidine (SO + Aminog). Panel D shows representative images of lung histology for the four experimental groups. Haematoxylin–eosin, high magnification (bar represents 20 µm). Each column represents mean ± SEM (n = 6). * p<0.05.

Treatment of obese mice with the selective iNOS inhibitor aminoguanidine (20 mg/kg, 3 weeks) fully restored the obesity-induced IR, as evaluated by the K_ITT_ values (Figure 8A). Aminoguanidine failed to affect the K_ITT_ values in lean group. In addition, aminoguanidine treatment significantly attenuated the eosinophil infiltration in the lung tissue of obese mice with concomitant normalization of eosinophil counts in BAL fluid of these animals (Figure 8B, C and D). In lean group, aminoguanidine significantly attenuated the eosinophil counts in BAL fluid with no significant effect on the eosinophil infiltration in the lung tissue.

### Effects of TNF-α and IL-5 Blocking on Airway Inflammatory Responses

**Figure 9:**
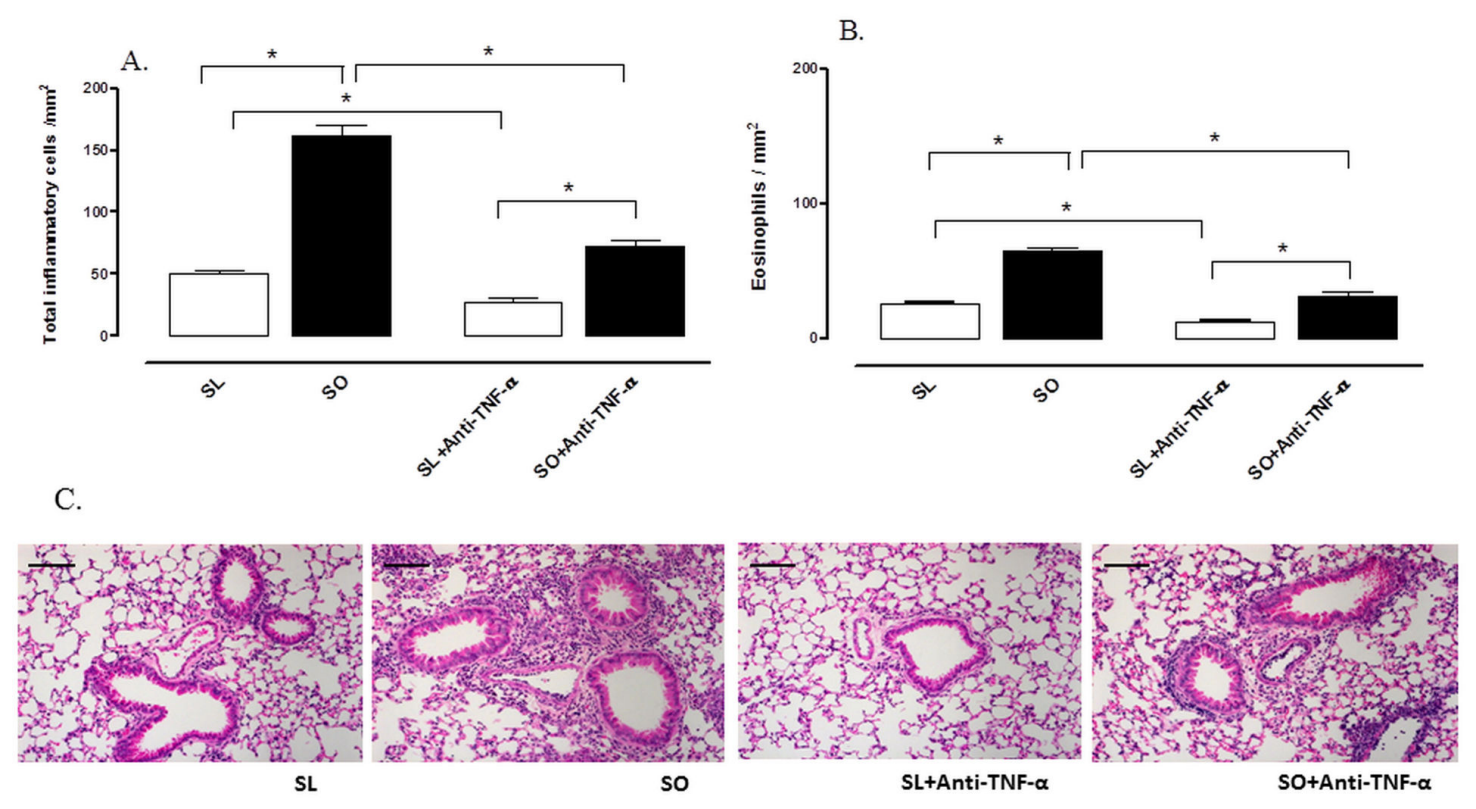
Effect of monoclonal anti-TNF-α antibody treatment (2 mg/kg) on the number of total inflammatory cells (A) and eosinophils (B) in lung connective tissue surrounding the bronchial and bronchiolar segments at 48 h following intranasal challenge with ovalbumin in sensitized mice. Anti-TNF-α antibody was given intraperitoneally at days 14 and 15 and 1 h before the first ovalbumin challenge. Representative high-power fields of bronchiolar structures from the following groups: sensitized lean (SL), sensitized obese (SO), sensitized lean treated with anti-TNF-α (SL + anti-TNF-α) and sensitized obese treated with anti-TNF-α (SO + anti-TNF-α). Panel C shows representative images of lung histology for the four experimental groups. Each column represents mean ± SEM (n = 6) of the number of cells mm^2^. Haematoxylin–eosin, high magnification (bar represents 20 µm). *p<0.05.

To evaluate the participation of TNF-α in the exacerbation of the eosinophilic inflammation in obese mice, we have treated animals with a neutralizing mouse mAb to TNF-α (2 mg/kg, i.p.). TNF-α blockade led to a marked reduction in the pulmonary infiltration of total cells and eosinophils in OVA-challenged obese mice compared with animals treated with the isotype IgG (Figure 9A, B and C). At the dose employed, anti-TNF-α also promoted significant inhibitions in the cell infiltration in lean group.

IL-5 plays an important role in the progression of the eosinophilic inflammatory response in allergic asthma. Treatment of animals with anti-IL-5 antibody (2 mg/kg, i.p.) did not affect the enhanced total cell and eosinophil infiltration in the lung of obese (171.7 ± 6.2 and 64.7 ± 3.8 cells/mm^2^, respectively) compared with animals receiving the isotype IgG at the same dose (188.4 ± 5.4 and 76.7 ± 7.4 cells/mm^2^, respectively). 

### Expression of Phosphorylated AMPK and Acetyl CoA Carboxylase (ACC) in the Lung Tissue

**Figure 10:**
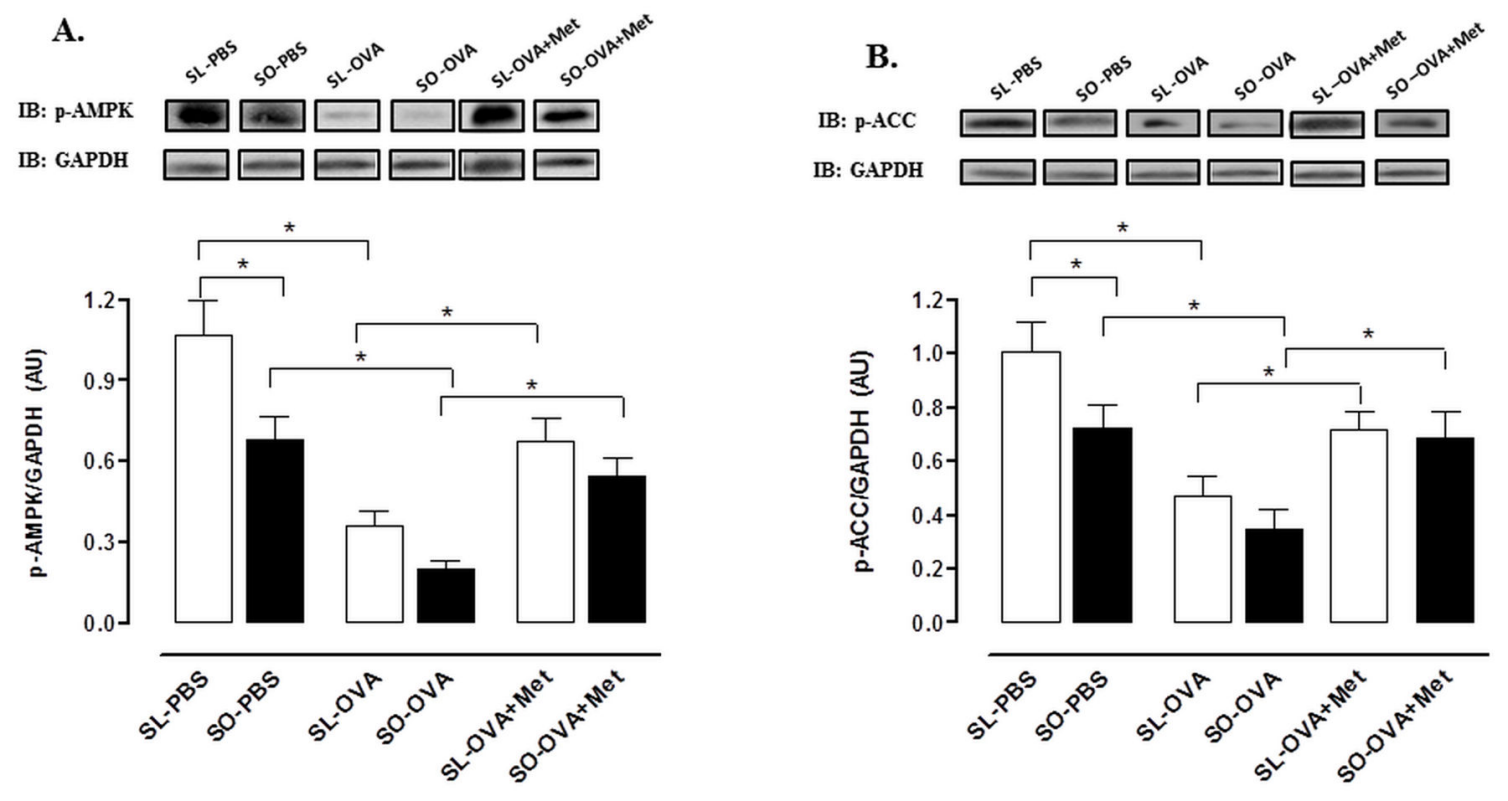
Effect of metformin treatment (300 mg/kg/day, two weeks) on phospho-AMPK (A) and phospho-acetyl CoA carboxylase (ACC) (B) expression in lung tissue at 48 h following intranasal challenge with ovalbumin in sensitized mice. Each column represents the mean ± SEM (n = 6) for the following groups of mice: sensitized lean treated with vehicle and instilled with PBS (SL-PBS), sensitized obese treated with vehicle and instilled with PBS (SO-PBS), sensitized lean treated with vehicle and challenged with OVA (SL-OVA), sensitized obese treated with vehicle and challenged with OVA (SO-OVA), sensitized lean treated with metformin and challenged with OVA (SL-OVA + Met) and sensitized obese treated with metformin and challenged with OVA (SO-OVA + Met). The membranes were normalized with GAPDH. * p<0.05.

Previous studies have implicated AMPK activation as a mediator of metformin action [37,38]. We therefore quantified the phosphorylation by metformin of AMPK (Thr 172 residue) and its downstream target ACC (Ser79 residue) in lung tissue of lean and obese. The basal levels of phosphorylated AMPK (PBS-instilled animals) were significantly lower in lung tissue of obese compared with lean mice (Figure 10A). Challenge with OVA reduced the levels of phosphorylated AMPK in both lean and obese groups, but the reduction was greater in the obese group. Metformin (300mg/kg/dia, 2 weeks) fully restored the levels of phosphorylated AMPK in the lung tissue of obese mice (Figure 10A). The levels of phosphorylated ACC in lung tissue at 48 h after OVA challenge were lower in obese compared with lean group, and that was also restored by metformin (Figure 10B).

## Discussion

This study shows that treatment of obese mice with the AMPK activator metformin suppressed IR and significantly reduced the OVA-induced eosinophil trafficking to the lung tissue, which was accompanied by reductions in the levels of eotaxin, TNF-α and NOx in BAL fluid. Metformin promoted marked decreases in the pulmonary iNOS expression and binding of NF-κB subunit (p65) to the iNOS promoter region in the lung tissue of obese mice. Metformin also elevated the levels of phosphorylated AMPK and its downstream target p-ACC in the lung tissue of obese mice indicating that AMPK activation may be a good pharmacological strategy to control the asthma exacerbation in obese individuals.

Obesity delays the eosinophil transit into the airway lumen, allowing these cells to remain longer in lung parenchyma [11]. Recent reports indicate a high prevalence of IR in obese and obese asthmatics *versus* non-asthmatics, suggesting that IR contributes to this phenotype [2,6]. In our study, treatment with metformin reduced the pulmonary eosinophil accumulation and restored the cell number in BAL fluid, suggesting that metformin facilitates the cell migration into the airway lumen, thus accelerating the clearance of tissue eosinophilia in obese mice. It is thus conceivable that amelioration of IR by metformin contributes to the pro-asthmatic phenotype in high-fat fed obese mice. 

Recent studies indicate AMPK loss favors a pro-inflammatory state [26]. In vitro activation of AMPK reduces cytokine production in the alveolar macrophage cell line MH-S and LTA-induced neutrophil influx, as well as the protein leakage and cytokine/chemokine levels in the bronchoalveolar space [39]. Autoimmune encephalomyelitis was more severe in AMPKα1^−/−^ mice than in controls [29] and knockdown of the gene that encodes AMPK in macrophages (or expression of a dominant negative mutant) promoted a pro-inflammatory state [31]. Our data showed that levels of phosphorylated AMPK (and its downstream target ACC) are decreased in the lung tissue of obese compared with lean mice, as detected in basal conditions (PBS groups) and after OVA challenge. Metformin treatment normalized the levels of phosphorylated-AMPK and p-ACC in the lung tissue of obese mice to the levels of lean group. This findings suggests that the beneficial effect of metformin on obese asthmatic mice may be at least partly dependent on AMPK activation, and that the AMPK signaling pathway could be an important regulator of eosinophilic inflammation in this pathological condition. In control (lean) mice challenged with OVA and fungal-associated allergenic protease (FAP), a recent study conducted by Park and col [40] showed that metformin reduces the number of inflammatory cells (especially eosinophils) in BAL fluid and enhances AMPK activation in the lung tissue. On the other hand, Shore et al [33] showed that metformin has no effect on airway inflammation and hyperresponsiveness induced by ozone in genetically obese mice. 

Eotaxins and IL-5 are involved in eosinophil-driven respiratory diseases [41]. Eotaxin attracts eosinophils with variable degrees of selectivity, and the presence and high levels of this chemokine in serum of asthmatic patients correlate with the severity of asthma disease [42]. Eotaxin acts conjointly with IL-5 in promoting eosinophil recruitment [43,44]. Increased levels and expression of eotaxin in serum and adipose tissue have also been shown in diet-induced obesity in mice and humans [45]. In our study, eotaxin-1 levels were greater in BAL fluid of obese mice that was restored by metformin treatment. However, the IL-5 levels remained elevated in metformin-treated obese mice. We decided next to examine the effects of neutralizing IL-5 with a mouse mAb in the eosinophilic airway inflammation in the obese mice. The pulmonary recruitment of total cells and eosinophils remained elevated by treatment with anti-IL-5 mAb, strongly suggesting that this cytokine does not play a role in the aggravation of the allergic eosinophilic inflammation in diet-induced obesity conditions. Development and test of humanized mAbs targeting human IL-5 (mepolizumab, reslizumab and benralizumab) in asthmatic individuals have resulted in controversial clinical outcomes [46,47]. Nevertheless, no study has explored the effects of such IL-5 mAbs in asthma exacerbations in obese individuals.

TNF-α acts directly in regulating adipocyte fat accumulation, interfering in glycemic homeostasis, lipid metabolism and IR [13,48]. Moreover, TNF-α plays an important role in the asthma physiopathology in humans and animals [49] acting as a chemotactic cytokine able to induce migration, proliferation and activation of leukocytes via a positive regulation of adhesion molecules expression such as VCAM-1 and ICAM-1, thus facilitating the cell migration [50,51]. TNF-α also induce the synthesis of eotaxin in different cell types [52,53]. It is thus plausible that the increased circulating levels of TNF-α in obesity contributes to the asthma exacerbations. TNF-α levels in BAL fluid were greater in obese mice and normalized after metformin treatment. Furthermore, treatment with an anti-TNF-α mAb largely reduced the allergic pulmonary eosinophilic inflammation in obese mice placing an important role for this cytokine in the asthma aggravation by obesity. According to literature [54], anti-TNF-α mAb has indeed a considerable anti-inflammatory effect on allergen-induced lung inflammation in lean mice.

Activation of AMPK by metformin reduces the activity of nuclear transcription factor NFκB induced by TNF-α in endothelial cells [55]. Therefore, metformin via inhibition of this transcriptional factor could inhibit the synthesis of various inflammatory mediators involved in asthma, obesity and association of both conditions. Among these pro-inflammatory mediators, iNOS-derived NO has been shown to play a major role in the allergic pulmonary eosinophilia in rodents [56]. Furthermore, the expression of iNOS in adipose tissue and skeletal muscle markedly increases after ingestion of hyperlipidic diet in mice [18]. Our data showing an increased iNOS expression in the lung tissue and consequently large amounts of NO in OVA-challenged obese mice strongly indicate that the iNOS-NO signaling contributes to the development of obesity-associated allergic diseases. This is reinforced by our findings that treatment with the selective iNOS inhibitor aminoguanidine reduced obesity-associated IR and OVA-induced eosinophilic inflammation in obese mice in a similar fashion to metformin. 

The levels of iNOS-derived NO in different cell types are mainly regulated at the transcriptional level. The promoter region of the mouse iNOS gene contains several binding sites for transcription factors, including NFkB. The regulation of iNOS via the NFkB pathway represents an important mechanism in inflammatory processes, and has a potential for intervention in pathological diseases [16]. Given the inhibitory effect of metformin on iNOS expression and NO overproduction, we next explored the mechanisms of iNOS inhibition in lungs of obese mice looking at the p65 NF-κB binding, using the Chip assay technique. Our results confirmed that metformin nearly abrogates the NFkB p65-binding to the iNOS promoter region in the lung tissue of obese mice, which may explain the reduced airways eosinophil infiltration in these animals. NO is reported to regulate its own production by exerting biphasic effects in its own synthesis (self-regulation) that takes place by inhibiting the expression of iNOS mRNA [57]. This mechanism may explain the reduced NFkB binding to the iNOS promoter region in obese mice at 48 h after OVA challenge.

TNF-α is an important regulator of granulocyte survival, mainly by activating the transcription factor NFκB [15]. Inhibition of NFκB in allergic pleurisy induces the resolution of eosinophilic inflammation due to increased apoptosis of these cells [58]. Exposure to TNF-α is likely to increase the activation of NF-κB and expression of iNOS. Conversely, reduction of TNF-α levels after metformin treatment in obese mice may lead to a decrease in NFκB activation and consequently to a decrease in anti-apoptotic factors, thereby facilitating the resolution of the inflammatory process. Accordingly, in obese mice, metformin reduced the OVA-induced eosinophil accumulation into the lung tissue and restored the cell number in BAL fluid to the levels of lean mice. This suggests that removal of granulocytes from airway tissues and egression into the airway lumen by metformin contribute to the resolution of airway inflammation. 

In conclusion, the reduction of pulmonary eosinophilia by metformin may be explained by the decreased production of NOx levels as consequence of inhibition of lung NFkB p65-binding to the iNOS promoter region, which fails to be activated by TNF-α. It is likely that obesity-associated IR contributes the exacerbation of pulmonary eosinophilic inflammation in high fat-diet mice. Activation of AMPK with metformin prevents the systemic IR, and animals respond to antigen challenge as sensitized lean mice accelerating the resolution of allergic airway inflammation. Drugs that control metabolic disorders secondary to obesity such as IR could become co-adjuvants in the treatment of asthma. 
